# TNF Signaling Pathway Is the Key Pathway Regulated by Disitamab Vedotin in Bladder Cancer Cells

**DOI:** 10.3390/cimb47050369

**Published:** 2025-05-18

**Authors:** Xingxing Tang, Jia Liu, Qiang Zhao, Yudong Cao, Xiao Yang, Peng Du, Yong Yang

**Affiliations:** Key Laboratory of Carcinogenesis and Translational Research (Ministry of Education), Department of Urology, Peking University Cancer Hospital & Institute, No. 52 Fucheng Road, Haidian District, Beijing 100142, China; xingxing.tang@bjcancer.org (X.T.); liujiauro@bjcancer.org (J.L.); pkuch_zhaoq@126.com (Q.Z.); 18611979333@126.com (X.Y.); yoya_urology@sina.com (Y.Y.)

**Keywords:** disitamab vedotin, RC48, bladder cancer, *TNF* signaling pathway, *NF-κB* signaling pathway

## Abstract

Disitamab Vedotin has shown good therapeutic efficacy against bladder cancer. Although its mechanism is clear, the regulation of gene expression in bladder cancer cells by Disitamab Vedotin is not fully understood. We searched the GEO database and identified the GSE237789 dataset, in which researchers treated the bladder cancer cell line SW780 with Disitamab Vedotin and performed high-throughput transcriptome sequencing. Compared with the control SW780 cells, the expression levels of the vast majority of genes (16,223/16,390, 98.98%) in Disitamab Vedotin-treated SW780 cells remained unchanged. Only one hundred fifty-nine genes (0.97%) were upregulated, and eight genes (0.05%) were downregulated. Enrichment analysis results showed that the related differentially expressed genes (DEGs) were mainly enriched in the *TNF* signaling pathway, *NF-κB* signaling pathway, and other pathways. Protein–protein interaction analysis revealed that 10 genes, *TNF*, *IL1B*, *IL1A*, *CXCL8*, *CXCL1*, *CCL2*, *MMP9*, *ICAM1*, *CXCL10*, and *CCL20*, had the highest connectivity, and all of these genes belong to the *TNF* signaling pathway. These results suggest that the *TNF* signaling pathway is the key pathway regulated by Disitamab Vedotin in bladder cancer cells, which may represent a stress response of bladder cancer cells to Disitamab Vedotin.

## 1. Introduction

Disitamab Vedotin (RC48) is an innovative antibody-drug conjugate (ADC) composed of a monoclonal antibody against human epidermal growth factor receptor 2 (HER2), a cleavable linker, and the cytotoxic agent Monomethyl Auristatin E (MMAE) [1]. The antibody component can recognize and bind to the surface of tumor cells with high HER2 expression. Subsequently, the drug enters the tumor cells and releases MMAE, which blocks the cell cycle process and induces cancer cell apoptosis [1]. Currently, this drug has demonstrated good therapeutic efficacy in several fields, including gastric cancer, breast cancer, cervical cancer, etc. [1,2,3,4].

In the field of urothelial carcinoma (UC), RC48 has also shown remarkable efficacy. The RC48-C005 study revealed that the objective response rate (ORR) in patients with HER2-positive locally advanced or metastatic UC reached 51.2% [5]. A combined analysis of the RC48-C005 and C009 studies indicated that among 107 HER2-positive patients with locally advanced or metastatic UC who received Disitamab Vedotin after first-line systemic chemotherapy failure, the ORR was 50.5%, the median progression-free survival (PFS) was 5.9 months, and the median overall survival (OS) was 14.2 months [6]. Another real-world study retrospectively collected data from 103 patients with metastatic UC treated with Disitamab Vedotin, showing an ORR of 50.5%, a disease control rate (DCR) of 79.6%, a median PFS of 6 months, and good safety [7]. The PUNCH02 study enrolled 10 patients with HER2 overexpression (IHC 2+ or 3+) in muscle-invasive bladder cancer (MIBC). These patients received combined treatment with tislelizumab and Disitamab Vedotin after transurethral resection of bladder tumor (TURBT). With an average follow-up of 5.7 months, the results showed a utDNA-defined clinical complete response (cCR) rate of 70.0%. Among patients with HER2 3+ and solitary tumors, the utDNA-defined cCR rate reached 100% [8]. Currently, both the FDA and China SFDA have approved Disitamab Vedotin for the treatment of UC [9].

Although the mechanism by which Disitamab Vedotin kills tumor cells, including bladder cancer cells, is clear, its regulation of gene expression in bladder cancer cells is not fully understood. With the increasing clinical use of Disitamab Vedotin, we believe that understanding its regulation of gene expression in bladder cancer cells is of great significance. Therefore, we utilized the publicly available Gene Expression Omnibus (GEO) database to collect relevant sequencing data and performed bioinformatics analysis to investigate the regulatory effects of Disitamab Vedotin on gene expression in bladder cancer cells.

## 2. Materials and Methods

### 2.1. Acquisition of Transcriptome Sequencing Data

We searched the GEO database using the keywords “bladder cancer”, “Disitamab Vedotin”, and “RC48” and identified the GSE237789 dataset (ncbi.nlm.nih.gov/geo/query/acc.cgi?acc=GSE237789 (accessed on 20 February 2025)), which is based on the BGISEQ-500 GPL23227 sequencing platform. This dataset includes six groups, with three groups serving as controls, in which the bladder cancer cells SW780 were untreated, and the other three groups representing the RC48 group, in which the SW780 cells were treated with Disitamab Vedotin (25 μg/mL) for 48 h. After drug treatment, RNA was harvested using TRIzol reagent, and 1.0 μg of total RNA was used for the construction of sequencing libraries. RNA libraries were prepared for sequencing following the standard BGI protocols. The dataset was provided by Li and colleagues from the Tianjin Institute of Urology and was uploaded on 1 June 2024. We used R (version 4.4.2) and the GEOquery package (version 2.74.0) to obtain the raw data of the GSE237789 dataset and perform the analysis.

### 2.2. Identification of Differentially Expressed Genes (DEGs)

We analyzed the DEGs between the RC48 group and the control group in the GSE237789 dataset using R (version 4.4.2) and the limma package (version 3.62.2). The Benjamini and Hochberg false discovery rate method was applied to calculate the adjusted *p*-values by default to reduce the false positive rate. Genes with adjusted *p* < 0.05 and log_2_(fold change) ≥ 1 or log_2_(fold change) ≤ −1 were designated as DEGs. The volcano plot was drawn using the SRplot online tools (bioinformatics.com.cn (accessed on 25 February 2025)) [10].

### 2.3. Gene Enrichment Analysis

Gene ontology (GO) analysis is a commonly used bioinformatics method for annotating genes and identifying biological characteristics of high-throughput transcriptomic data [11]. The Kyoto Encyclopedia of Genes and Genomes (KEGG) is an open database containing information on biological signaling pathways of genes [12]. We performed online enrichment analysis of DEGs, using the g:Profiler website (biit.cs.ut.ee/gprofiler (accessed on 27 February 2025)) to clarify the GO functions and KEGG pathway enrichment of DEGs [13], and visualized the biological processes (BP), molecular functions (MF), cellular components (CC), and KEGG pathway enrichment results of these DEGs using the SRplot online tools. The cutoff criteria were set at *p* < 0.05 and false discovery rate (FDR) < 0.05.

### 2.4. Protein–Protein Interaction (PPI) Network Analysis and Identification of Hub Genes

The Search Tool for the Retrieval of Interacting Genes (STRING) is an important online tool for evaluating PPI network information [14]. We used the STRING database (cn.string-db.org (accessed on 28 February 2025), version 12.0) to assess the potential PPI relationships among the relevant DEGs and then constructed and visualized the PPI network of these DEGs using Cytoscape software (version 3.10.3). The degree of each DEG was calculated, and the top 10 genes with the highest degree were defined as hub genes.

### 2.5. Correlation Analysis of Hub Gene Expression

Based on the expression levels of hub genes in the six samples of the two groups mentioned above, we performed gene expression correlation analysis using the Pearson correlation coefficient method to determine whether there is a correlation in expression levels between any two hub genes. The visualization of correlation analysis was conducted using the SRplot online tools, with *p* < 0.05 indicating statistical significance.

### 2.6. Identification of Signaling Pathways for Hub Genes

The visualization of the location of hub genes in the enriched KEGG signaling pathways was performed using the SRplot online tools (bioinformatics.com.cn (accessed on 28 February 2025)).

### 2.7. Cell Culture

The human bladder cancer cell lines SW780 and 5637 were obtained from the Key Laboratory of Carcinogenesis and Translational Research at Peking University. The 5637 cells were cultured in Dulbecco’s Modified Eagle Medium (DMEM) containing 10% fetal bovine serum (FBS) and 1% penicillin/streptomycin, while the SW780 cells were cultured in Roswell Park Memorial Institute (RPMI) 1640 medium containing 10% FBS and 1% penicillin/streptomycin. All cells were cultured in a humidified incubator at 37 °C with 5% CO_2_. Both SW780 and 5637 cells were divided into two groups: the control group was treated with 0.1% dimethyl sulfoxide (DMSO), and the Disitamab Vedotin group was treated with 25 μg/mL Disitamab Vedotin. All cells were treated for 48 h.

### 2.8. Main Reagents and Instruments

The RPMI 1640 medium (12633020) was purchased from Thermo Fisher (Waltham, MA, USA). The DMEM (C11995500BT) was purchased from Thermo Fisher. The PBS was purchased from HyClone (Logan, UT, USA). The FBS (04-001-1ACS) was purchased from BI (Wheeling, IL, USA). The penicillin/streptomycin antibiotic (15140-122) was purchased from Life Technologies (Carlsbad, CA, USA). Disitamab Vedotin (CAS No. 2136633-23-1) was purchased from Selleck (Houston, TX, USA). The TRIzol reagent (15596-018) was purchased from Invitrogen (Carlsbad, CA, USA). The cDNA Reverse Transcription Kit (4368813) was purchased from Applied Biosystems (Foster, CA, USA). The GoTaq qPCR Master Mix (A6001) was purchased from Promega (Madison, WI, USA). The Real-Time PCR system (7500 Fast) was purchased from Applied Biosystems.

### 2.9. Quantitative PCR (qPCR)

Total RNA was extracted from SW780 and 5637 cells using TRIzol reagent. cDNA was synthesized from 1 μg RNA using the high-capacity cDNA Reverse Transcription Kit. qPCR was then performed using GoTaq qPCR Master Mix and the Applied Biosystems 7500 Fast Real-Time PCR system to determine the mRNA expression levels of the target genes. The expression levels of all target genes were normalized to the housekeeping gene GAPDH. The primer sequences were as follows: for *TNF*, forward primer CCTCTCTCTAATCAGCCCTCTG and reverse primer GAGGACCTGGGAGTAGATGAG; for *IL1B*, forward primer ATGATGGCTTATTACAGTGGCAA and reverse primer GTCGGAGATTCGTAGCTGGA; for *IL1A*, forward primer AGATGCCTGAGATACCCAAAACC and reverse primer CCAAGCACACCCAGTAGTCT; and for *GAPDH*, forward primer GGAGCGAGATCCCTCCAAAAT and reverse primer GGCTGTTGTCATACTTCTCATGG. The primer sequences for the remaining genes are not listed in detail.

### 2.10. Statistics

The statistical analysis of qPCR data and the plotting of related images were performed using GraphPad Prism v9.3.1 (GraphPad Software, San Diego, CA, USA). All data are presented as mean ± standard deviation. Data comparisons among control and Disitamab Vedotin groups were conducted using a *t*-test, and a *p*-value of less than 0.05 was considered to indicate a statistically significant difference.

## 3. Results

### 3.1. DEGs Regulated by Disitamab Vedotin in Bladder Cancer Cells

**Figure 1:**
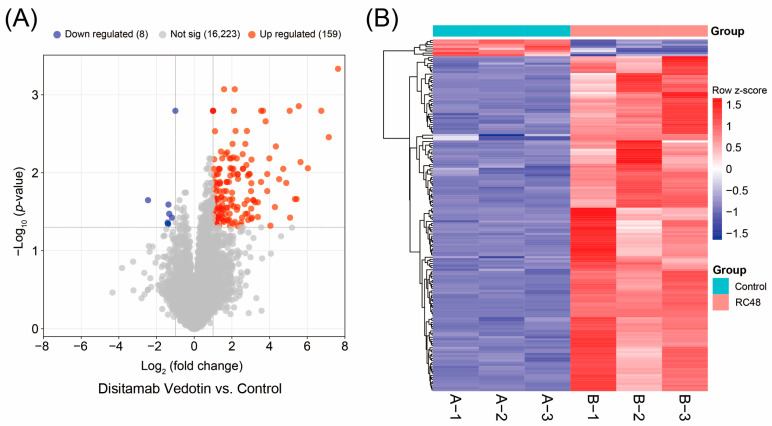
DEGs between RC48-treated SW780 cells and control cells. (**A**) Volcano plot of gene expression differences between RC48-treated SW780 cells and control group cells, with red representing upregulated genes, blue representing downregulated genes, and gray representing genes with no significant difference in expression. (**B**) Heatmap of DEGs between the two groups of cells, with red indicating upregulation and blue indicating downregulation. A-1, A-2, A-3, B-1, B-2, and B-3 are numbering of the six groups of cells for transcriptome analysis, with A representing the control group and B representing the Disitamab Vedotin-treated group. Not sig, not significant.

The GSE237789 dataset includes six groups of data, with three groups serving as controls, i.e., the untreated bladder cancer cell line SW780 cells, and the other three groups as the RC48 group, i.e., SW780 cells treated with Disitamab Vedotin. The dataset is based on the GPL23227 BGISEQ-500 (*Homo sapiens*) platform, and RNA was harvested using TRIzol reagent. A total of 1.0 μg of RNA was used for the construction of sequencing libraries. DEGs were defined as genes with an upregulation or downregulation fold change exceeding 2, i.e., log_2_(fold change) ≥ 1 or log_2_(fold change) ≤ −1, and an adjusted *p*-value < 0.05. The results showed that compared with the control SW780 cells, the expression levels of the vast majority of genes (16,223/16,390, 98.98%) in the RC48 group remained unchanged, with only a small number of genes (167/16,390, 1.02%) showing significant changes in expression. Among these, the majority of genes (159/167, 95.21%) were upregulated, while only a very small number of genes (8/167, 4.79%) were downregulated (Figure 1A). The gene expression heatmap also indicated that the regulation of gene expression in bladder cancer cells by Disitamab Vedotin was predominantly characterized by upregulation (Figure 1B). The top fifteen upregulated DEGs and the top eight downregulated DEGs are listed in Table 1. Since there were only eight downregulated genes, subsequent analyses focused solely on the upregulated genes.

### 3.2. GO Function and KEGG Pathway Enrichment Analyses of DEGs

**Figure 2:**
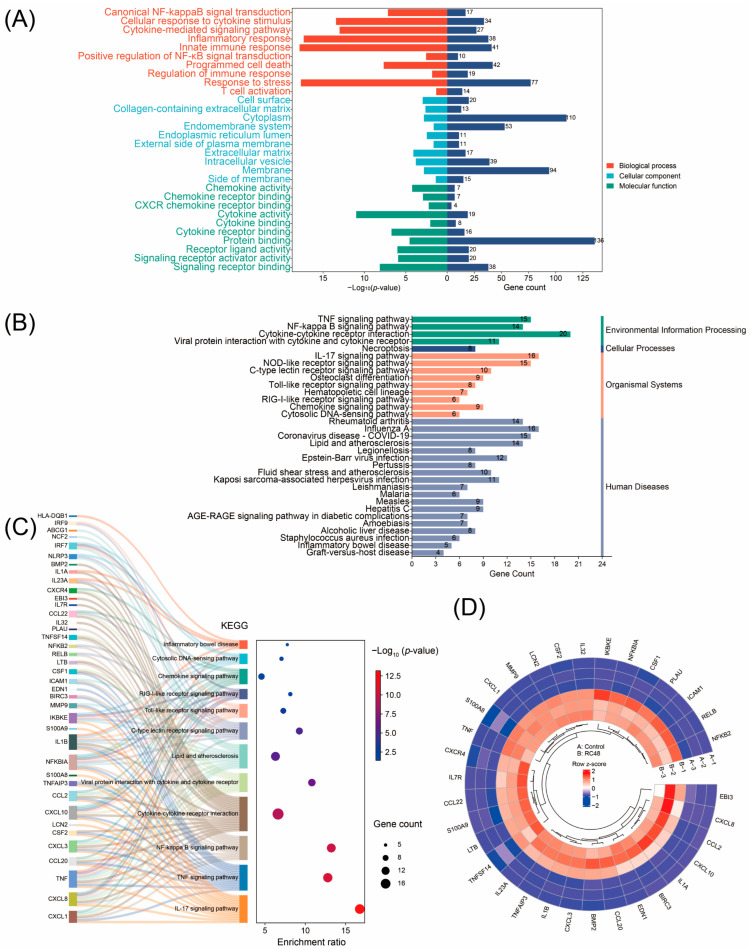
Enrichment of DEGs between Disitamab Vedotin-treated SW780 cells and control cells. (**A**) The GO enrichment results of DEGs in RC48-treated SW780 cells compared with control cells; all the enriched signaling pathways were upregulated in cells treated with Disitamab Vedotin compared with the control cells. (**B**) The KEGG pathway enrichment results of DEGs in the two groups of cells; all the enriched signaling pathways were upregulated in cells treated with Disitamab Vedotin compared with the control cells. (**C**) The genes corresponding to each enriched pathway, with the size of the circle representing the number of enriched genes. (**D**) The expression difference heatmap of the 33 DEGs enriched in KEGG pathways among all 159 DEGs, in both control and Disitamab Vedotin-treated groups. A-1, A-2, A-3, B-1, B-2, and B-3 are numbering of the six groups of cells for transcriptome analysis, with A representing the control group and B representing the Disitamab Vedotin-treated group.

Gene ontology (GO) enrichment analysis revealed that the upregulated DEGs were primarily enriched in biological processes such as canonical *NF-κB* signal transduction (Figure 2A). KEGG pathway enrichment analysis showed that the upregulated DEGs were mainly enriched in pathways such as the *NF-κB* signaling pathway and the *TNF* signaling pathway (Figure 2B). The KEGG pathway analysis of DEGs associated with bladder cancer cells is presented in Table 2. Further analysis of the key pathways enriched by KEGG and their corresponding DEGs indicated that the *IL-17* signaling pathway, *TNF* signaling pathway, *NF-κB* signaling pathway, and cytokine–cytokine receptor interaction pathway had the lowest FDR and contained the largest number of DEGs. DEGs such as *TNF*, *NFKBIA*, *IL1B*, and *CXCL8* were involved in the greatest number of pathways (Figure 2C). Among the 159 upregulated DEGs, 33 DEGs were enriched in these pathways, and the heatmap analysis intuitively displayed the differences in the expression levels of these DEGs between the two groups of cells (Figure 2D). The above analyses revealed that the regulation of gene expression in bladder cancer cells by Disitamab Vedotin mainly focused on the *IL-17* signaling pathway, *TNF* signaling pathway, *NF-κB* signaling pathway, and cytokine–cytokine receptor interaction pathway.

### 3.3. Identification of Hub Genes Through PPI Network Analysis

**Figure 3:**
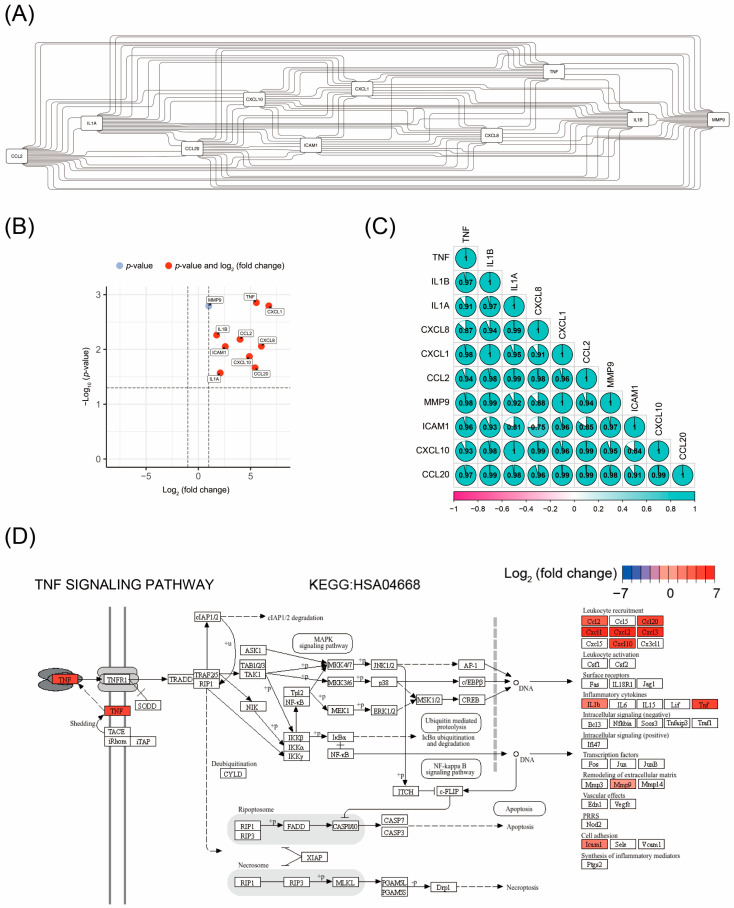
Protein interaction analyses of the DEGs between RC48-treated SW780 cells and control cells. (**A**) Hub gene PPI network diagram, where the lines connecting genes indicate that the proteins transcribed by these two genes have interaction information in the STRING database. (**B**) Hub gene expression volcano plot, with red representing genes that have significant fold changes and *p*-values and blue representing genes with only significant *p*-values for expression differences. (**C**) Hub gene expression correlation diagram, where the data inside the circle and the angle of the opening represent the correlation coefficient, and the green color indicates positive correlation. (**D**) The distribution and upstream/downstream relationships of hub genes in the *TNF* signaling pathway (KEGG: HSA04668), with red indicating upregulation of gene expression in RC48-treated cells and the intensity of the red color representing the degree of upregulation.

Using the STRING PPI analysis database, we further conducted PPI network analysis on the DEGs related to the main pathways identified in the enrichment analysis. The results showed that 10 DEGs, *TNF*, *IL1B*, *IL1A*, *CXCL8*, *CXCL1*, *CCL2*, *MMP9*, *ICAM1*, *CXCL10*, and *CCL20*, had the highest connectivity (Figure 3A) and were all located within the enriched pathways. Among them, *CXCL1*, *CXCL8*, and *TNF* exhibited the highest fold changes, indicating the greatest differences in expression levels between the control and RC48 groups (Figure 3B). The top 10 hub genes with the highest degrees of connectivity are listed in Table 3.

### 3.4. Correlation Analysis of the Expression Levels of Hub Gene

We performed correlation analysis on the expression levels of the 10 hub genes, *TNF*, *IL1B*, *IL1A*, *CXCL8*, *CXCL1*, *CCL2*, *MMP9*, *ICAM1*, *CXCL10*, and *CCL20*. The results showed that, except for the lack of correlation between *ICAM1* and *IL1A* and between *ICAM1* and *CXCL8*, the expression levels of all other pairs of genes were significantly correlated (*p* < 0.05) (Figure 3C).

### 3.5. TNF Signaling Pathway as the Key Pathway Regulated by Disitamab Vedotin in Bladder Cancer Cells

By matching the above 10 hub genes with information from the KEGG signaling pathway database, we found that the *TNF* signaling pathway included all these 10 hub genes and also encompassed the *NF-κB* signaling pathway, which was enriched in the pathways (Figure 3D).

### 3.6. TNF Signaling Pathway Genes Are Upregulated in Bladder Cancer Cells Treated with Disitamab Vedotin

**Figure 4:**
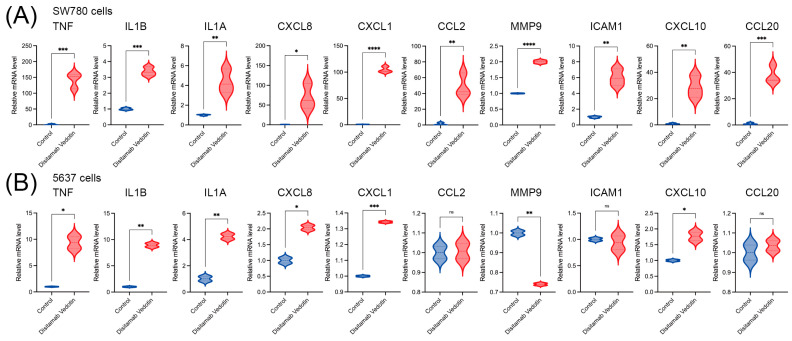
The regulation of Disitamab Vedotin on hub gene expression (mRNA level) in bladder cancer cells was analyzed by qPCR. (**A**) Violin plot of relative mRNA level of the hub genes of control and Disitamab Vedotin-treated SW780 cells. (**B**) Violin plot of relative mRNA level of the hub genes of control and Disitamab Vedotin-treated 5637 cells. *, *p* < 0.05; **, *p* < 0.01; ***, *p* < 0.001; ****, *p* < 0.0001; ns, no significance.

The qPCR results showed that, compared to the control group, the expression levels of all ten hub genes (at the mRNA level) in the Disitamab Vedotin group were significantly upregulated in SW780 cells (all *p* < 0.05), including *TNF*, *IL1A*, *IL1B*, *CXCL8*, *CXCL1*, *CCL2*, *MMP9*, *ICAM1*, *CXCL10*, and *CCL20* (Figure 4A). Similar results were observed in 5637 cells as well, where the expression levels of *TNF*, *IL1A*, *IL1B*, *CXCL8*, *CXCL1*, and *CXCL10* were significantly upregulated in the Disitamab Vedotin group compared to the control group (all *p* < 0.05), but there was no significant difference in the expression of *CCL2*, *ICAM1,* and *CCL20* between the control and Disitamab Vedotin groups in 5637 cells. Moreover, the expression level of *MMP9* in the Disitamab Vedotin-treated 5637 cells was significantly lower than that in the control group (*p* < 0.01) (Figure 4B). Overall, these results suggest the regulation of the *TNF* signaling pathway by Disitamab Vedotin at the mRNA level in bladder cancer cells and also indicate that different bladder cancer cell lines may respond slightly differently to Disitamab Vedotin.

Combining all the above analyses, we conclude that the *TNF* signaling pathway is the key pathway regulated by Disitamab Vedotin in bladder cancer cells.

## 4. Discussion

Disitamab Vedotin, as a novel ADC, is composed of a humanized anti-HER2 monoclonal antibody conjugated to MMAE via a linker [1,15]. The mechanism of Disitamab Vedotin is well defined: it can target and kill tumor cells under the guidance of HER2. In addition to its efficacy in cancers such as gastric and breast cancer [3,4], it has also demonstrated good efficacy and safety in the treatment of UC [5,6,7]. Multiple studies have shown that the ORR in patients with HER2-positive locally advanced or metastatic UC treated with Disitamab Vedotin is around 50% [5,6].

Although the mechanism by which Disitamab Vedotin kills tumor cells is clear, its regulation of gene expression in bladder cancer cells is not fully understood. To address this, we searched the GEO database and identified the only relevant dataset, GSE237789, published by Li and colleagues from the Tianjin Institute of Urology. The researchers divided the bladder cancer cell line SW780 into two groups: a control group and an RC48 group. The control group cells were left untreated, while the RC48 group cells were treated with Disitamab Vedotin. After treatment, total RNA was extracted from each group of cells and subjected to transcriptome sequencing to observe the regulation of mRNA levels and gene expression in bladder cancer cells by Disitamab Vedotin. Using this dataset, our transcriptomic analysis revealed that, after treatment with Disitamab Vedotin, the expression levels of the vast majority of genes (16,223/16,390, 98.98%) remained unchanged, with only a small number of genes (167/16,390, 1.02%) showing significant changes. Among these, the majority (159/167, 95.21%) were upregulated, while only a very small number (8/167, 4.79%) were downregulated. Given the minimal number of downregulated genes, we focused our analysis on the upregulated genes. Further enrichment analysis of these upregulated DEGs showed that most could be enriched in the *IL-17* signaling pathway, *TNF* signaling pathway, *NF-κB* signaling pathway, and cytokine–cytokine receptor interaction pathway. A total of 33 DEGs were involved in these enriched pathways. Subsequent PPI analysis indicated that among these 33 DEGs, 10 genes—*TNF*, *IL1B*, *IL1A*, *CXCL8*, *CXCL1*, *CCL2*, *MMP9*, *ICAM1*, *CXCL10*, and *CCL20*—had the highest connectivity and thus played the most central roles in these pathways. We then matched these 10 hub genes with information from the KEGG signaling pathway database and found that the *TNF* signaling pathway included the majority of these hub genes. Considering all the above factors, we concluded that the *TNF* signaling pathway is the key pathway regulated by Disitamab Vedotin in bladder cancer cells. It is worth noting that since the activation of the *TNF* signaling pathway is closely related to the *NF-κB* signaling pathway, which was also identified in the enrichment results, the *NF-κB* signaling pathway is also one of the main pathways regulated by Disitamab Vedotin.

Starting from the above-mentioned hub genes, we comprehensively analyzed the potential role of *TNF* signaling pathway activation in bladder cancer cells exposed to Disitamab Vedotin. Inflammatory factors such as *TNF* and *IL-1* can bind to their respective receptors and phosphorylate IκB kinase. The latter, after ubiquitination, can be recognized and degraded by the 26S proteasome. Consequently, *NF-κB* is released from the cytoplasmic *NF-κB/IκB* complex, exposing its nuclear localization domain and forming a *P50/RelA* dimer that translocates into the nucleus [16]. Subsequently, *NF-κB* can bind to the κB response elements of target genes via its P50 subunit, thereby initiating target gene expression [17]. In this process, *TNF* can activate *NF-κB*, while *NF-κB* can also regulate the expression of the *TNF* gene, forming an interactive network between the two [18]. *IL1B* and *IL1A*, as important inflammation-related cytokines, have their gene expression regulated by *NF-κB*. During inflammatory responses, *NF-κB* activation promotes the secretion of *IL1B* and *IL1A* [19]. The gene promoters of chemokines *CXCL8*, *CXCL1*, *CCL2*, *CXCL10*, and *CCL20* contain binding sites for *NF-κB*. After *NF-κB* activation, it can bind to these sites, promoting the transcription and expression of chemokines, thereby recruiting immune cells to the site of inflammation [20,21]. *MMP9*, which is involved in processes such as extracellular matrix degradation, also has its gene expression regulated by *NF-κB*. In various pathological conditions, including inflammation and cancer, *NF-κB* can regulate cell migration and invasion by modulating *MMP9* expression [22]. *ICAM1*, as an adhesion molecule, is also a target gene of *NF-κB*. *NF-κB* can upregulate *ICAM1* expression, enhancing intercellular adhesion, which facilitates the adhesion of immune cells to endothelial cells and promotes immune cell infiltration into the site of inflammation [23]. These results suggest that when bladder cancer cells are exposed to Disitamab Vedotin, the upregulation of *TNF*, *IL1B*, and *IL1A*, followed by receptor binding, activates the *NF-κB* signaling pathway. This, in turn, increases the expression of *CXCL8*, *CXCL1*, *CCL2*, *CXCL10*, and *CCL20*. These chemokines can recruit immune cells and trigger local inflammatory responses. Additionally, the activated *NF-κB* signaling pathway can also transcribe *MMP9* and *ICAM1*, enhancing extracellular matrix degradation and intercellular adhesion. These changes in gene expression may represent a stress response of bladder cancer cells to Disitamab Vedotin, potentially aiding in the proliferation and survival of cancer cells in the presence of Disitamab Vedotin.

This study has some notable limitations. First, we only conducted bioinformatics analysis and qPCR without performing histological, cytological, or animal experiments to validate the regulation of the *TNF* signaling pathway in bladder cancer cells by Disitamab Vedotin in vivo and in vitro. Second, the sample size of the dataset we used was small, with only three samples in each group, and increasing the sample size would make our conclusions more convincing. Additionally, we did not further explore the function and significance of *TNF* signaling pathway activation in bladder cancer cells exposed to Disitamab Vedotin, which could be a direction for future research.

## 5. Conclusions

In summary, the results of these bioinformatics analyses indicate that the *TNF* signaling pathway is the key pathway regulated by Disitamab Vedotin in bladder cancer cells. This may represent a stress response of bladder cancer cells to Disitamab Vedotin, potentially aiding in the proliferation and survival of cancer cells in the presence of Disitamab Vedotin. However, considering the limitations of this study, further studies are needed.

## Figures and Tables

**Table 1:** Top DEGs between control and Disitamab Vedotin-treated bladder cancer cells.

Top 15 Upregulated DEGs *				
**Gene Symbol**	**Log_2_ (Fold Change)**	**Adjusted** ***p*-Value**	***t***	**B**
*UBD*	8.974	0.010	14.425	3.185
*LTB*	7.633	0.000	65.409	7.323
*ERCC5*	7.143	0.003	22.952	5.079
*CXCL1*	6.744	0.002	28.700	5.801
*CXCL8*	6.032	0.009	16.185	3.696
*IL32*	5.643	0.007	17.179	3.951
*TNF*	5.549	0.001	38.924	6.560
*CCL20*	5.434	0.022	10.857	1.843
*CXCL3*	5.349	0.022	10.923	1.873
*CSF2*	5.087	0.038	8.334	0.535
*LCN2*	5.068	0.002	28.913	5.822
*CXCL10*	4.887	0.014	12.956	2.690
*KLHDC7B*	4.725	0.009	15.411	3.481
*SAA1*	4.508	0.012	13.646	2.931
*C3*	4.334	0.005	21.047	4.763
**Top 8 Downregulated DEGs**				
**Gene Symbol**	**Log_2_ (Fold Change)**	**Adjusted *p*-Value**	***t***	**B**
*HOTS*	−2.451	0.022	−10.652	1.750
*FAM81A*	−1.408	0.044	−7.787	0.196
*ZNF334*	−1.390	0.045	−7.669	0.120
*MEGF10*	−1.390	0.045	−7.669	0.120
*CLCA2*	−1.368	0.026	−10.030	1.455
*RGS16*	−1.327	0.034	−8.814	0.814
*GJA1*	−1.184	0.038	−8.320	0.527
*UGT2B28*	−1.000	0.002	−28.944	5.826

* DEGs, differentially expressed genes.

**Table 2:** KEGG pathway analysis of DEGs * associated with Disitamab Vedotin treatment.

Source	Term Name	FDR *	Count	Intersections
KEGG	*IL-17* signaling pathway	<0.001	16	*CXCL1*, *CXCL8*, *TNF*, *CCL20*, *CXCL3*, *CSF2*, *LCN2*, *CXCL10*, *CCL2*, *TNFAIP3*, *S100A8*, *NFKBIA*, *IL1B*, *S100A9*, *IKBKE*, *MMP9*
KEGG	*TNF* signaling pathway	<0.001	15	*CXCL1*, *TNF*, *CCL20*, *CXCL3*, *CSF2*, *CXCL10*, *CCL2*, *TNFAIP3*, *BIRC3*, *EDN1*, *ICAM1*, *NFKBIA*, *IL1B*, *CSF1*, *MMP9*
KEGG	*NF-kappa B* signaling pathway	<0.001	14	*LTB*, *CXCL1*, *CXCL8*, *TNF*, *CXCL3*, *TNFAIP3*, *BIRC3*, *RELB*, *ICAM1*, *NFKB2*, *TNFSF14*, *NFKBIA*, *IL1B*, *PLAU*
KEGG	Cytokine-cytokine receptor interaction	<0.001	20	*LTB*, *CXCL1*, *CXCL8*, *IL32*, *TNF*, *CCL20*, *CXCL3*, *CSF2*, *CXCL10*, *CCL2*, *CCL22*, *IL7R*, *EBI3*, *CXCR4*, *IL23A*, *IL1A*, *TNFSF14*, *IL1B*, *BMP2*, *CSF1*
KEGG	Viral protein interaction with cytokine and cytokine receptor	<0.001	11	*CXCL1*, *CXCL8*, *TNF*, *CCL20*, *CXCL3*, *CXCL10*, *CCL2*, *CCL22*, *CXCR4*, *TNFSF14*, *CSF1*
KEGG	Lipid and atherosclerosis	<0.001	14	*CXCL1*, *CXCL8*, *TNF*, *CXCL3*, *CCL2*, *ICAM1*, *NFKBIA*, *NLRP3*, *IL1B*, *IRF7*, *IKBKE*, *NCF2*, *ABCG1*, *MMP9*
KEGG	C-type lectin receptor signaling pathway	<0.001	10	*TNF*, *CCL22*, *RELB*, *IL23A*, *NFKB2*, *NFKBIA*, *NLRP3*, *IL1B*, *IRF9*, *IKBKE*
KEGG	Toll-like receptor signaling pathway	0.001	8	*CXCL8*, *TNF*, *CXCL10*, *NFKBIA*, *IL1B*, *IRF9*, *IRF7*, *IKBKE*
KEGG	RIG-I-like receptor signaling pathway	0.008	6	*CXCL8*, *TNF*, *CXCL10*, *NFKBIA*, *IRF7*, *IKBKE*
KEGG	Chemokine signaling pathway	0.013	9	*CXCL1*, *CXCL8*, *CCL20*, *CXCL3*, *CXCL10*, *CCL2*, *CCL22*, *CXCR4*, *NFKBIA*
KEGG	Cytosolic DNA-sensing pathway	0.018	6	*CXCL10*, *NFKBIA*, *NLRP3*, *IL1B*, *IRF7*, *IKBKE*
KEGG	Inflammatory bowel disease	0.040	5	*TNF*, *IL23A*, *IL1A*, *IL1B*, *HLA-DQB1*

* DEGs, differentially expressed genes; FDR, false discovery rate.

**Table 3:** Top 10 hub genes with the highest degrees of connectivity.

Rank	Gene Symbol	Gene Description	Degree	Log_2_ (Fold Change)	Adjusted *p*-Value
1	*TNF*	Tumor Necrosis Factor	62	5.549	0.001
2	*IL1B*	Interleukin 1 Beta	60	1.752	0.005
3	*IL1A*	Interleukin 1 Alpha	58	2.094	0.027
4	*CXCL8*	C-X-C Motif Chemokine Ligand 8	58	6.032	0.009
5	*CXCL1*	C-X-C Motif Chemokine Ligand 1	54	6.744	0.002
6	*CCL2*	C-C Motif Chemokine Ligand 2	50	3.989	0.007
7	*MMP9*	Matrix Metallopeptidase 9	50	1.000	0.002
8	*ICAM1*	Intercellular Adhesion Molecule 1	50	2.580	0.009
9	*CXCL10*	C-X-C Motif Chemokine Ligand 10	50	4.887	0.014
10	*CCL20*	C-C Motif Chemokine Ligand 20	48	5.434	0.022

## Data Availability

All the datasets generated in this study are available on reasonable request to the corresponding author.
